# Tumor suppressor genes in the tumor microenvironment

**DOI:** 10.1242/dmm.052049

**Published:** 2025-03-20

**Authors:** Bahareh Tabanifar, Hannah Lau, Kanaga Sabapathy

**Affiliations:** ^1^ Division of Cellular & Molecular Research, National Cancer Centre Singapore, Singapore 168583; ^2^ Department of Physiology, National University of Singapore, Singapore 117558; ^3^ School of Biological Sciences, Nanyang Technological University, Singapore 637551

**Keywords:** Fibroblasts, TP53, PTEN, Tumor microenvironment, Tumor suppressors

## Abstract

Tumor suppressor genes (TSGs) are thought to suppress tumor development primarily via cancer cell-autonomous mechanisms. However, the tumor microenvironment (TME) also significantly influences tumorigenesis. In this context, a role for TSGs in the various cell types of the TME in regulating tumor growth is emerging. Indeed, expression analyses of TSGs in clinical samples, along with data from mouse models in which TSGs were deleted selectively in the TME, indicate a functional role for them in tumor development. In this Perspective, using *TP53* and *PTEN* as examples, we posit that TSGs play a significant role in cells of the TME in regulating tumor development, and postulate both a ‘pro-active’ and ‘reactive’ model for their contribution to tumor growth, dependent on the temporal sequence of initiating events. Finally, we discuss the need to consider a 2-in-1 cancer-treatment strategy to improve the efficacy of clearance of cancer cells and the cancer-promoting TME.

## Teaser

**Summary:** The role of tumor suppressors within the tumor microenvironment; with particular focus on TP53 and PTEN, and their roles in non-cell autonomous regulation of tumor development.

## Introduction

Oncogene activation and tumor suppressor gene (TSG; Box 1) inactivation are considered to be the primary causal events in the transformation of normal cells *en route* to malignancy (Solomon et al., 2010; Hanahan and Weinberg, 2011). Alterations in their expression are often sufficient to promote cellular proliferation or inhibit apoptosis, eventually resulting in cellular transformation, thereby underscoring their cell-autonomous roles in the tumorigenic process (Hanahan and Weinberg, 2011; Zhu et al., 2015). Not surprisingly, most cancer genome sequencing efforts have focused on cataloguing such alterations, and molecular therapies target these tumor-cell-specific alterations (Guo et al., 2014). However, it is increasingly clear that the tumor microenvironment (TME; Box 1) that surrounds tumor cells (Anderson and Simon, 2020) impacts on tumor initiation, progression, metastasis and therapy resistance (Valkenburg et al., 2018; de Visser and Joyce, 2023). Recent studies have also alluded to a significant role for adipocytes and myofibroblasts in prognosticating overall survival in breast cancers (Li et al., 2021; Lau et al., 2023). Interestingly, modifications in stromal gene expression, especially of TSGs, have been postulated to affect neighboring tumor cells (Moinfar et al., 2000; Scherz-Shouval et al., 2014; Wu et al., 2022). For instance, loss of tumor suppressor functions in the stroma (Box 1) may either directly affect tumor cell properties or, alternatively, lead to pre-malignant changes in the adjacent epithelial cells (Bhowmick et al., 2004; Wu et al., 2022), although these effects could be cell-type specific. Whether the expression and functions of oncogenes and TSGs in the stromal cells (Box 1) of the TME play a significant role in the tumorigenic process has not been unequivocally established. We focus here on the role of two commonly mutated TSGs in the TME, i.e. *TP53* and *PTEN* (Table 1; Box 2), and highlight their significant contribution to the tumor development process. Since fibroblasts are the most abundant cell type in the TME, most studies have investigated the role of *TP53* and *PTEN* in these cells, as a representative of the TME. Although *TP53* and *PTEN* are the most extensively researched TSGs in this context, similar but limited findings have been made with other TSGs, such as *RB1* (Mercier et al., 2008; Pickard et al., 2012; Kitajima et al., 2020).

**Box 1. Tumor microenvironment**

**Cancer-associated fibroblasts (CAFs):** Fibroblasts or cells with mesenchymal-like features that are the most common cell type of TME in solid tumors. Their presence is often associated with poor prognosis, resistance to therapies, and disease recurrence.

**Stroma:** Supporting or connective tissue consisting of mesenchymal cells such as fibroblasts, immune cells, vasculature network, extracellular matrix, pericytes and adipocytes. In this Perspective, stroma refers to the niche surrounding the epithelial cells or tumor cells. In some cases, the stromal element is specified like stromal fibroblast. Otherwise, stroma refers to the whole compartment around the epithelial or tumor cells.

**Stromal cells of TME:** Major class of cellular components in TME including fibroblasts, mesenchymal stem cells, adipocytes, endothelial cells, pericytes, osteoblasts and astrocytes.

**Tumor-associated stroma:** Stromal niche around tumor cells.

**Tumor microenvironment (TME):** Complex cellular system that surrounds a tumor assisting its growth and expansion. It comprises the non-malignant cellular and non-cellular components, including immune cells, stromal cells, extracellular matrix, blood vessels, lymphatic vessels, cytokines and other non-cellular components.

**Tumor suppressor genes (TSGs):** Genes that prevent cells from growing uncontrollably and becoming cancerous.

**Box 2. Glossary**

**APC^min^-driven colon cancer model:** Multiple intestinal neoplasia (min), is a mutation in the murine adenomatous polyposis coli (APC) gene that when engineered in a mouse model predisposes the animals to colorectal and intestinal tumors.

**APR-246 (also known as eprenetapopt):** Small molecule that reactivates mutant TP53 and selectively induces apoptosis in *TP53*-mutant cancer cells.

**ATM (ataxia telangiectasia mutated):** Serine/threonine protein kinase that is involved in the repair of double-strand DNA breaks. Mutations in ATM are associated with increased cancer development.

**ATR (ataxia telangiectasia and Rad3-related):** Serine/threonine protein kinase that is active during the S phase of the cell cycle and orchestrates the response to replication stress to maintain genome integrity.

**ERBB2 (erb-b2 receptor tyrosine kinase 2; also known as HER2, NEU):** Receptor tyrosine-protein kinase that controls cell growth and division.

**Kras^G12D^:** *Kras* proto-oncogene with a mutation in codon 12. The GTPase KRAS plays role in cell proliferation and survival.

**Carbon tetrachloride-induced liver cancer model:** Continual administration of carbon tetrachloride results in hepatocyte injury that eventually leads to liver cancer development.

**Lysyl oxidases:** Family of enzymes that are involved in shaping the structure of the extracellular matrix. Their enzymatic activity is also associated with regulation of many cellular functions including proliferation, survival and differentiation.

**MCF-7:** Human breast cancer cell line that comprises estrogen, progesterone and glucocorticoid receptors.

**miR-21:** MicroRNA (miRNA)-21 is one of the most abundant microRNAs in many mammalian cells. It is an oncogenic miRNA that targets many tumor suppressors. MicroRNAs are short non-coding RNA molecules that regulate eukaryotic gene expression at the post-transcriptional level.

**PARP (poly [ADP-ribose] polymerase 1):** DNA-binding enzyme involved in the DNA-repair pathway for single strand breaks.

**PLK1 (polo-like kinase 1):** PLKs are a family of serine/threonine protein kinases. PLK1 plays an important role in the initiation, maintenance and completion of mitosis. Overexpression of *PLK1* has been found in a variety of human cancers.

**TP53 (tumor protein p53):** Well-characterized tumor suppressor protein that is encoded by *TP53*, the most frequently mutated gene in human cancers.

**PTEN (phosphatase and tensin homolog):** Protein phosphatase with tumor suppressor activity. PTEN plays role in cell growth and survival and is commonly mutated or deleted in different types of cancers.

**PTEN−miR-320−ETS2 axis:** A regulatory axis discovered in the stroma of breast cancer tissue, where PTEN regulates the expression of *mir-320* microRNA (miRNA-320). miR-320 consequently regulates gene expression of the *ETS2* transcription factor. This axis is shown to regulate fibroblast secretome.

**WEE1 (WEE1 G2 checkpoint kinase):** Member of a serine/threonine protein kinases, causing G2-M cell-cycle arrest in response to DNA damage.

**Synthetic lethality:** Phenomenon whereby the concurrent deficiency of two genes results in cellular death, whereas deficiency of one individual gene alone does not cause cell death. Synthetic lethality is one of the most effective treatment strategies in cancer therapy.

**Y220C mutant:** Hot spot mutation in codon 220 of *TP53* that results in decreased DNA binding and reduced TP53 transcriptional activity.

**Table 1.** Consequences of *TP53* and *PTEN* inactivation in different cells of the stroma on tumor growth

Inactivation of *TP53* within the stroma			
Cell type with *TP53* deletion/mutation	Effect on tumor	Type of cancer	References
MEFs	supportive	Prostate, breast	Addadi et al., 2010; Kiaris et al., 2005; Lafkas et al., 2008; Liu et al., 2020
Fibroblasts	supportive	Breast	Wu et al., 2022
CAFs	suppressive	Lung	Arandkar et al., 2018
Hepatic stellate cells	supportive	Liver	Lujambio et al., 2013
Microphages	supportive	Colorectal	He et al., 2015

Inactivation of *PTEN* within the stroma			
Cell type with *PTEN* deletion	Effect on tumor	Type of cancer	References
Fibroblasts	supportive	Prostate, breast	Trimboli et al., 2009
Macrophages	supportive	Pancreatic	Wang et al., 2018
Endothelial	supportive	Lung, melanoma	Hamada et al., 2005
Tregs	suppressive	Lung, melanoma, lymphoma	Sharma et al., 2015

CAFs, cancer-associated fibroblasts; MEFs, mouse embryonic fibroblasts; Treg, regulatory T cells.

## Alteration of TSG expression in the TME

Several studies have demonstrated differential gene expression in tumor-associated stroma (Box 1) relative to normal stroma (Finak et al., 2008; Bauer et al., 2010; Navab et al., 2011). These differential expressions are associated with alterations, such as loss of heterozygosity, microsatellite instability and mutations (Eng et al., 2009), in a variety of solid tumors, including breast (Moinfar et al., 2000; Kurose et al., 2002), ovarian (Tuhkanen et al., 2004), head and neck (Bian et al., 2007), and bladder cancers (Paterson et al., 2003). For instance, frequent somatic mutations are found in *TP53* and *PTEN* in the stroma of invasive breast carcinomas (Kurose et al., 2002). Besides genetic alterations, epigenetic changes − such as histone modification and miRNA regulation − also control gene expression in the stroma (Marks et al., 2016; Yang et al., 2023), such as in cancer-associated fibroblasts (CAFs; Box 1) (Vizoso et al., 2015; Xiao et al., 2016), immune cells (Janson et al., 2008) and endothelial cells (Chung et al., 2007). For example, stromal PTEN has been shown to be regulated by both hypermethylation and miRNA (Bian et al., 2012; Yang et al., 2014). In this Perspective, we will discuss in more detail the influence alterations in *TP53* and *PTEN* within the TME have on tumor development.

### TP53

*TP53* is mutated or lost in >50% of solid tumors (Sabapathy and Lane, 2018) and is also mutated in the stromal compartment (Kurose et al., 2002; Hill et al., 2005; Patocs et al., 2007). For example, TP53 protein expression and function are often suppressed in CAFs (Hawsawi et al., 2008; Bar et al., 2009) and suppression of *TP53* gene expression in stromal fibroblasts induces their transition to CAFs (Procopio et al., 2015). Silencing *TP53* gene expression in cultured fibroblasts increased production of stromal cell-derived factor 1 (SDF-1, officially known as CXCL12) in these cells and promoted the migration and invasion of leukemic and osteosarcoma cells *in vitro* (Moskovits et al., 2006). Furthermore, co-injection of the human prostate epithelial cancer cell line PC3 with fibroblasts of different *TP53* status into xenograft models also showed that fibroblast-specific loss of TP53 expression promotes tumor growth and metastasis in an SDF-1 dependent manner (Addadi et al., 2010). Likewise, the presence of mutant TP53 in fibroblasts promoted prostate cancer cell growth and metastasis (Liu et al., 2020). Similar findings were noted when using the human breast cancer cell line MCF-7 (Box 2) (Kiaris et al., 2005). However, *TP53*-null fibroblasts sensitize tumor cells to chemotherapeutic agents, such as doxorubicin and cisplatin (Lafkas et al., 2008), and radiotherapy (Burdelya et al., 2006), indicating that stromal TP53 also influences the response to therapy.

Consistent with the *ex vivo* findings, selective deletion of *TP53* in fibroblasts, together with the expression of mutated oncogene *Kras^G12D^* (Box 2) in the epithelium, promoted the formation of mammary tumors in genetically modified mice by creating an immunosuppressive TME (Wu et al., 2022). This effect was not observed when mammary epithelial cells expressed the oncogene *ErbB2* (Box 2) (Wu et al., 2022), indicating that the effects of ablation in stromal TP53 occur in an oncogene-specific manner. In particular, loss of *TP53* in fibroblasts led to the reprogramming of gene expression in not only the fibroblasts but, more interestingly, in the adjacent epithelial cells (Wu et al., 2022), supporting the idea that loss of tumor suppressor functions in the TME is sufficient to promote genetic alteration in neighboring tumor cells. Similar results have been noted with *TP53* deletion in other cell types of the TME, such as macrophages (He et al., 2015) or hepatic stellate cells (Lujambio et al., 2013), in an APC^min^-driven colon cancer model (Box 2) or a carbon-tetrachloride-induced liver cancer model (Box 2), respectively.

Contradictory to the established tumor-suppressive role of TP53, an intriguing observation indicated that TP53 function is required to maintain the tumor-promoting phenotype induced by CAFs, in which the secretome is significantly altered by TP53. In this case, CAF TP53 is re-wired to become tumor-supportive, as its depletion in CAFs significantly reduced lung-tumor growth in a xenograft model (Arandkar et al., 2018). This finding highlights a different function of TP53 in naive versus activated fibroblasts, where not only tumor-suppressive functionality of TP53 is quenched but TP53 undergoes alterations that confer it a tumor-supportive function.

### PTEN

*PTEN* is frequently disrupted in sporadic tumors and has profound impacts on tumor progression (Thies et al., 2019). Besides tumor cells, loss of *PTEN* gene expression has been reported in the stroma of breast (Kurose et al., 2002; Trimboli et al., 2009), prostate (Ashida et al., 2012) and pancreatic cancers (Pitarresi et al., 2018), and is associated with worse outcome in the patients. Immunohistochemical staining has shown that PTEN is lost in stroma of ∼25% of breast cancer patients (Trimboli et al., 2009). Indeed, loss of heterozygosity and point mutations of *PTEN* have been reported in stromal fibroblasts of breast cancers (Kurose et al., 2001, 2002). Similarly, loss of PTEN expression has been reported in 20% of stromal fibroblasts of pancreatic cancer samples associated with hemizygous deletion of *PTEN* and chromosome 10 monosomy (Wartenberg et al., 2016). Besides genetic alterations, upregulation of miR-21 (Box 2) was reported as another mechanism leading to loss of PTEN protein expression in the collective stromal compartment of pancreatic cancer (Wartenberg et al., 2016).

As well as the above observations in patients, several studies have modeled PTEN loss in the stroma and investigated the impact on tumor progression. *PTEN* knockdown in patient-derived CAFs accelerated human pancreatic ductal adenocarcinoma tumor cell growth in an orthotopic co-injection mouse model (Pitarresi et al., 2018). Conditional knockout of *PTEN* in fibroblasts of a mouse breast cancer model (*MMTV-ErbB2*) altered the expression profile of these cells and accelerated initiation and progression of mammary tumor (Trimboli et al., 2009). Mechanistically, deletion of *PTEN* in stromal fibroblasts led to increased extracellular matrix deposition and innate immune cell infiltration via the PTEN−miR-320−ETS2 axis (Box 2) (Trimboli et al., 2009; Bronisz et al., 2011).

*PTEN* deletion in fibroblasts has also been shown to affect adjacent epithelial cells and lead to the expansion of mammary epithelial stem cell enriched populations (Sizemore et al., 2017). This occurs together with the downregulation of the DNA-repair response in the adjacent mammary epithelial cells, thereby promoting genome instability in the epithelial compartment (Sizemore et al., 2018). These effects on mammary epithelial cells, although insufficient to drive tumorigenesis, increase the risk of breast cancer initiation after a second carcinogenic hit (Sizemore et al., 2018). Similarly, tissue-specific deletion of *PTEN* in transgenic mice showed that collective stromal PTEN expression restrains endometrial carcinogenesis in mice in which *PTEN* has been deleted within uterine epithelial (Liang et al., 2018).

*PTEN* deletion in other components of the TME also impacts tumorigenesis. In macrophages, loss of PTEN induced a pro-tumorigenic M2 polarization, which led to increased pancreatic cell metastasis to lung (Wang et al., 2018). Furthermore, heterozygous *PTEN* deficiency in endothelial cells of transgenic mice enhanced tumor angiogenesis in melanoma and lung tumors (Hamada et al., 2005).

In contrast to the studies highlighted above, in which stromal PTEN has an inhibitory impact on tumor progression, PTEN expression in regulatory T cells has been shown to stabilize them within the tumor and maintain an immunosuppressive TME (Sharma et al., 2015). In this case, *PTEN* deletion in regulatory T cells reduces the growth of lung cancer, melanoma and lymphoma. Therefore, stromal PTEN has cell-type specific functions in tumor suppression and, surprisingly, tumor progression.

## A ‘pro-active’ and ‘reactive’ role for TSGs in the TME

**Fig. 1.**
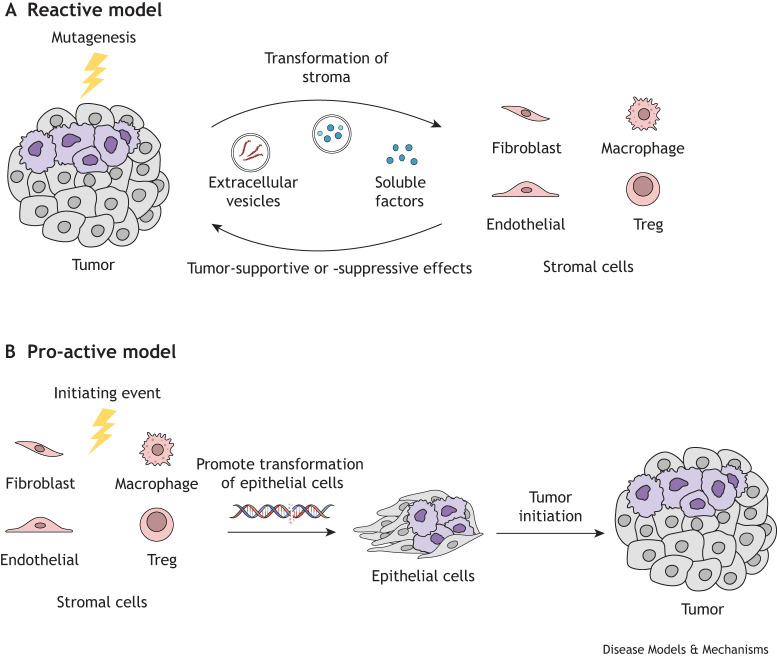
**A ‘pro-active’ and ‘reactive’ role for TSGs in TME.** In the ‘reactive model’, malignant tumor cells initiate a series of events that lead to the transformation and conversion of the stroma (via extracellular vesicles, soluble factors, etc.) to inactivate the TSGs in the cells of the stroma. This is a reaction from the stroma to the presence of the tumor cells, which then generally supports the growth of the tumor. In the ‘pro-active’ model, the initiating event is the inactivation of the TSG within the stromal cells (through germline mutations, epigenetic changes, carcinogenic signals, transient effects by metabolites, etc.), which then provides the signals to initiate changes that lead to the transformation of neighboring epithelial cells. Once initiated, the epithelial cells undergo transformation and continue their growth through the reactive model. Both models differ in the initiating event and, thus, are dictated by the temporal sequence of events. Treg, regulatory T cell.

The currently available literature indicates that, in most cell types of the stroma, TSG inactivation promotes tumor development. However, whether this is a pro-active initiating event or a reactive consequence of the tumor milieu, is yet to be unequivocally established (Fig. 1). Whereas the reactive model is easier to envision because of DNA-damage or carcinogen-induced cellular transformation that then affects the stroma, the pro-active model is probably due to changes in gradients induced by metabolites that may then affect TSG activity (Kong et al., 2024). As experimental validation of both contexts to establish a temporal series of events is currently limited, future work will shed light on support for these models.

Available literature provides arguments in favor of the possibility of both contexts of TSG inactivation in stromal cells: either in response to the oncogenic stress imposed by adjacent tumor cells resulting in a strong selective pressure for a TSG-deficient stroma during tumor progression or, alternatively, in the pro-active context by loss of TSGs in stromal cells that may precede their loss in tumor cells

Available literature provides arguments in favor of the possibility of both contexts of TSG inactivation in stromal cells: either in response to the oncogenic stress imposed by adjacent tumor cells resulting in a strong selective pressure for a TSG-deficient stroma during tumor progression or, alternatively, in the pro-active context by loss of TSGs in stromal cells that may precede their loss in tumor cells (Palumbo et al., 2015). In the reactive model, it can be envisaged that the initial presence of a pre-cancerous lesion leads to the transformation of the surrounding stroma through shuttling of oncogenic miRNAs − or even mutant proteins, such as mutant TP53 − via extra-cellular vesicles (Cooks et al., 2018; Bhatta et al., 2021) that, in turn, facilitate the malignant growth. Significant evidence for such influence of the tumor on the stromal transcriptome has been demonstrated (Aran et al., 2017; Lau et al., 2023). In the pro-active model, a permissive TME with reduced TSG activity can also be a fertile ground for easier transformation of embedded epithelial cells, as often seen in the case of familial cancers (Moinfar et al., 2000; Brosseau and Le, 2019). For example, somatic *TP53* mutations in stromal fibroblasts have been shown to increase genome instability in adjacent epithelia and are associated with breast cancer and increased regional nodal metastasis (Moinfar et al., 2000; Patocs et al., 2007).

Long-term organoid co-culture models could be exploited to examine both possibilities experimentally, with the genetic manipulation of either the epithelial cells or stromal fibroblasts, together with single-cell genomic and transcriptomic analyses. Overall, we propose that both pro-active and reactive models are likely to be operational, depending on the contexts and initiating events.

Given the key alterations in the TSGs of TME cells, it is imperative that they are considered during cancer management […]. Targeting the malignant cells alone is insufficient to completely eradicate the disease.

## Implications in cancer therapy

Given the key alterations in the TSGs of TME cells, it is imperative that they are considered during cancer management, which is not the current clinical practice especially with molecular targeted therapies. Targeting the malignant cells alone is insufficient to completely eradicate the disease. Thus, a 2-in-1 approach of simultaneously targeting both the tumor and the stroma should be considered. To achieve that, more work needs to be done to establish the alterations in the TME, as has been done for the malignant cells. This will set the stage for molecular targeting of the TME. Given the continuing advances in genomic approaches, single-cell RNA and DNA analysis can provide the necessary information on the altered TSG and related pathways that can then be therapeutically targeted in the TME.

Currently, loss of TSG function, regardless of the cell type, is difficult to restore therapeutically. However, several approaches have been proposed to target inactivated TSGs, including synthetic lethality (Box 2) to induce death in cells with inactivated TSGs, as well as compounds that could restore functionality of the mutant protein. In the former case, synthetic lethal interactions between loss of TP53 function and inhibition of ATR (Box 2) (Reaper et al., 2011), or inhibition of PLK1 (Box 2) (Meng et al., 2013) or inhibition WEE1 (Box 2) (Bridges et al., 2011), amongst others have been noted. Similarly, loss of PTEN activity was synthetically lethal when coinciding with inhibition of PARP (Box 2) (Mendes-Pereira et al., 2009), ATM (Box 2) (McCabe et al., 2015) or lysyl oxidases (Box 2) (Chen et al., 2019), amongst others. As such, some of these inhibitors could be used in combination with both regular cancer therapy and inhibitors that target specific alterations found in cancer cells. In the latter, compounds that might restore functionality in mutant TSGs have been identified, primarily targeting mutant TP53. Some of them − such as APR-246 (Box 2) that targets many TP53 mutants, and rezatapopt that specifically targets the Y220C mutant TP53 (Box 2) − were shown to have efficacy in pre-clinical studies (Synnott et al., 2018; Dumbrava et al., 2022; Carter et al., 2023). These drugs could be combined with cancer treatment protocols if the molecular signatures of the TME alterations are known.

More effort is required to identify and classify factors and drugs that significantly impact TSG alterations. The combination of the ability to detect and the availability of drugs to target the TSG alterations is necessary to develop 2-in-1 approach during which both the tumor and TME cells are targeted concomitantly. This, we think is likely to result in eradication of cancer in its entirety.

TME is a key contributor to tumor development and significantly influences therapeutic response. […] TSGs have a significant contributory role in TME alteration and, consequently, tumor properties.

## Concluding remarks

It is becoming increasingly evident that the TME is a key contributor to tumor development and significantly influences therapeutic response. By using *TP53* and *PTEN* as prominent examples, this *Perspective* has highlighted that TSGs have a significant contributory role in TME alteration and, consequently, tumor properties. We also envisage that similar alterations in other TSGs, such as *RB1*, and oncogenes will contribute significantly to the altered TME. Thus, establishing whether alterations in TSGs (and oncogenes) in the TME are a prevalent and universal phenomenon in cancers is a critical first step in furthering efforts to target cancer for therapy. Determining the contexts in which the stroma can be a pro-active component that promotes tumorigenesis will allow the development of potential prophylactic strategies that slow down or even prevent the progression of malignancies.
